# Regulation of Phosphorylated State of NMDA Receptor by STEP_61_ Phosphatase after Mild-Traumatic Brain Injury: Role of Oxidative Stress

**DOI:** 10.3390/antiox10101575

**Published:** 2021-10-05

**Authors:** Francisco J. Carvajal, Waldo Cerpa

**Affiliations:** 1 Laboratorio de Función y Patología Neuronal, Centro de Envejecimiento y Regeneración (CARE), Departamento de Biología Celular y Molecular, Facultad de Ciencias Biológicas, Pontificia Universidad Católica de Chile, Santiago 8331150, Chile; francisco.carvajalc@outlook.cl; 2 Centro de Excelencia en Biomedicina de Magallanes (CEBIMA), Universidad de Magallanes, Punta Arenas 6200000, Chile

**Keywords:** mild traumatic brain injury, STEP_61_, SOD2^+/−^ mice, oxidative stress, synaptic transmission, hippocampus, NMDARs, behavioral performance

## Abstract

Traumatic Brain Injury (TBI) mediates neuronal death through several events involving many molecular pathways, including the glutamate-mediated excitotoxicity for excessive stimulation of N-methyl-D-aspartate receptors (NMDARs), producing activation of death signaling pathways. However, the contribution of NMDARs (distribution and signaling-associated to the distribution) remains incompletely understood. We propose a critical role of STEP_61_ (Striatal-Enriched protein tyrosine phosphatase) in TBI; this phosphatase regulates the dephosphorylated state of the GluN2B subunit through two pathways: by direct dephosphorylation of tyrosine-1472 and indirectly via dephosphorylation and inactivation of Fyn kinase. We previously demonstrated oxidative stress’s contribution to NMDAR signaling and distribution using SOD2^+/−^ mice such a model. We performed TBI protocol using a controlled frontal impact device using C57BL/6 mice and SOD2^+/−^ animals. After TBI, we found alterations in cognitive performance, NMDAR-dependent synaptic function (decreased synaptic form of NMDARs and decreased synaptic current NMDAR-dependent), and increased STEP_61_ activity. These changes are reduced partially with the STEP_61_-inhibitor TC-2153 treatment in mice subjected to TBI protocol. This study contributes with evidence about the role of STEP_61_ in the neuropathological progression after TBI and also the alteration in their activity, such as an early biomarker of synaptic damage in traumatic lesions.

## 1. Introduction

Traumatic brain injury (TBI) is a structural and physiological disruption of brain function resulting from an external force. It is one of the leading causes of death and disability in industrialized countries [1]. TBI is a multifaceted disorder. In the initial stage, patients suffer from surface contusion, focal damage, and intracranial hemorrhages. This primary damage immediately generates damage to the brain, making a possible window of clinical/therapeutic intervention unfeasible.

On the other hand, these primary injuries can trigger more secondary severe effects such as neuroinflammation, oxidative stress, bioenergetic impairment, and alterations of endogenous neurochemical mechanisms [2,3,4]; these secondary damage events can occur from hours to months after trauma events, offering an advantageous therapeutic window for intervention. The hippocampal injury associated with learning and memory deficits are the frequent hallmarks of brain trauma and are the most enduring and devastating consequences of TBI [5]. The hippocampus undergoes similar neuropathological changes after both human closed-head injury and experimental injury models of TBI [6,7]. In this case, we studied the effect of frontal impact close-head injury, the most frequent cause of TBI [8], on the hippocampal glutamatergic transmission and the functional consequences in a murine model of mild repetitive TBI, focusing our attention on the regulation and functionality of N-methyl-D-aspartate receptors (NMDARs).

The modulation of the dynamics of glutamate receptors has grown in complexity in recent years, mainly due to the important discoveries related to the movement of NMDA-like receptors between synaptic and extrasynaptic zones [9,10]. NMDARs participate in signaling cascades that depend on their location. Synaptic NMDARs signal towards survival processes, whereas NMDARs located in the extrasynaptic are related to apoptotic signaling [11,12]. The activation of these extrasynaptic receptors can contribute to cognitive dysfunction and the appearance and development of neurodegenerative diseases such as Huntington’s disease (HD) and Alzheimer’s disease (AD) [13,14]. An essential component of synaptic NMDAR signaling includes the transcriptional factor CREB (through phosphorylation of serine 133) related to neuronal survival [15]. Conversely, the activation of extrasynaptic NMDARs induces the shut-off of CREB signaling (by dephosphorylation) [12], and at the same time activates pro-apoptotic signaling cascades [16,17,18,19].

It has recently been established that the lateral movement of NMDARs between synaptic and extrasynaptic sites is an essential component of the dynamics of these receptors [11,20]. A series of cellular components participate in this dynamic, including the cytoskeletal, scaffolding proteins and signaling complexes, and direct posttranslational modifications on NMDARs [9,21]. The main posttranslational modification that regulates the traffic of NMDARs corresponds to the phosphorylation of specific residues. Phosphorylation at tyrosine 1472 in the GluN2B subunit of NMDARs is characteristic of their synaptic location [22]. On the other hand, phosphorylation at tyrosine 1336 in the GluN2B subunit is associated with a place in extrasynaptic zones of NMDARs [23]. Thus, regulation of these phosphorylation sites in GluN2B occurs at different levels.

On the one hand, both sites are the substrate of the Fyn kinase (Src-family) [22]. On the other hand, STEP_61_ phosphatase directly dephosphorylates the GluN2B-Tyr 1472 site [23,24]. But this phosphatase also inactivates the Fyn protein by dephosphorylation [25].

Additionally, STEP_61_ is differentially regulated by synaptic and extrasynaptic NMDARs [26]. Finally, STEP_61_ is proteolytically cleaved by calpain [26], generating STEP_33_, a truncated and inactive STEP_61_ product.

The substrate of STEP_61_, p38, is activated after extrasynaptic NMDARs stimulation through proteolytic cleavage of STEP_61_ initiates the cell death signaling cascade [27]. Part of this mechanism could involve the etiology of neurodegenerative disorders linked to oxidative stress, such as AD, HD, and schizophrenia, where STEP_61_ is central [17,28,29]. We previously demonstrated that mice with low antioxidant capacity (SOD2^+/−^) show alterations in the synaptic/extrasynaptic ratio of GluN2B.

We previously demonstrated that mice with low antioxidant capacity (SOD2^+/−^) show alterations in the synaptic / extrasynaptic relationship of GluN2B and an increase in STEP_61_ protein levels in animals older that presenting higher levels of oxidative stress markers [19]. The mechanism that relates oxidative stress to modulation of STEP_61_ activity involves forming dimers and oligomers of STEP_61_ [30]. With the finality of corroborated the role of oxidative stress in STEP_61_ activity and their correlation with synaptic and cognitive failure after TBI, we evaluated if SOD2 suppression further aggravates brain damage in a model of mild and repetitive TBI. This study’s heterozygous SOD2^+/−^ mice exhibit decreased SOD2 activity in several tissues [31]. SOD2 activity in the brain of 5-week-old SOD2^+/−^ animals is at least 79% lower than in WT mice of the same age [31]. We evaluated the behavioral performance and synaptic transmission under oxidative stress and TBI conditions and focused on the downstream signaling of NMDARs activation. We found impairments in learning and memory processes and alterations in glutamatergic synaptic transmission, mainly in the NMDARs response after TBI in WT and SOD2^+/−^ mice. We found increased extrasynaptic NMDARs signaling and changes in the molecular machinery implicated in the distribution of these receptors. The levels of STEP_61_ phosphatase increase, lower the synaptic form of NMDA receptors; and decreased levels of serine-133-phosphorylated CREB, a transcriptional factor activated by the synaptic response. STEP_61_-inhibitor TC-2153 treatment can partially reduce the effects of TBI.

Therapeutic approaches that specifically rectify receptor mislocalization or target resulting downstream apoptotic signaling could be beneficial for preventing disease onset or progression across many disorders commonly caused by NMDAR dysfunction as occurs after TBI.

## 2. Materials and Methods

### 2.1. Animals

Heterozygous Sod2tm1Leb/J mice (SOD2^+/−^) were bred from SOD2^+/−^ breeding pairs obtained from The Jackson Laboratory (Sacramento, CA). The colony was established by crossing SOD2^+/−^ with C57BL/6J wild-type mice (WT, used as control animals). Genotyping was performed using a protocols database from Jax Labs. All the male animals, WT and SOD2^+/−^ have two-month-old at the start of the TBI induction session and were housed in temperature and light-controlled rooms, with food and water ad libitum in the Animal Facility of the Facultad de Ciencias Biológicas, Pontificia Universidad Católica de Chile. The animals were administered intraperitoneal (IP) injections of TC-2153 (10 mg/Kg), dissolved in DMSO (dimethyl sulfoxide; see Supplementary Figure S1C). Control treatments included IP injections of DMSO. All the procedures were approved by the Pontificia Universidad Católica de Chile bioethics committee, registration number 180814017.

### 2.2. Chemicals and Antibodies

The primary antibodies used are listed in Supplementary Table S1. All secondary antibodies used were obtained from Jackson Immunoresearch, Baltimore, USA (dilution 1:200). Chemicals used: 2,3-Dioxo-6-nitro-1,2,3,4-tetrahydrobenzo[f]quinoxaline-7-sulfonamide disodium salt (NBQX, Tocris, Bristol, UK), Picrotoxin (PTX, Tocris, Bristol, UK), and TC-2153 (benzopentathiepin 8-(trifluoromethyl)-1,2,3,4,5-benzopentathiepin-6-amine hydrochloride, Sigma-Aldrich, St. Louis, MO, USA).

### 2.3. Traumatic Brain Injury Induction Protocol

The frontal impact injury device was modified from Kilbourne, 2009 [32]. This device consists of two parallel rails (two pieces of 8mm-diameter tubular steel, joined 3 cm apart at their center). The impact energy was applied via a steel ball (180 g) accelerated by gravity by rolling down from a vertical height of 1 m (Supplementary Figure S1A). The end of the rails held at a 60° angle from a horizontal by a pedestal, curved gently into horizontally directed ends, thus redirecting the ball’s trajectory horizontally. At the ends of its run, the rolling ball struck a coupling arm. This arm is on a partition that carries the animal from a direct hit of the ball. Therefore, the coupling arm is engaging in the malar processes. This arm consists of a solid cylinder (diameter, 25 mm, and 50 mm long). The coupling arm has two rods. One rod protruded 3 mm farther than the other rod. When the coupling arm was struck, the 3-mm offset induced sagittal rotational acceleration of the head, in addition to axial acceleration. When assembled and affixed to the snout, the coupling arm protruded 2 cm from the tip of the snout, thereby allowing a horizontally directed frontal impact without damaging the animal’s facial structures.

The protocol of repetitive TBI induction consists of five sessions every two days. Each session consisted of 3 consecutive blasts per day. As previously described in [33,34], more details are in supplementary methods (Supplementary Figure S1B). Animals were anesthetized in an isoflurane chamber.

### 2.4. Immunohistochemical Procedures

Perfusion, fixation, and free-floating immunofluorescence procedures were performed in 30 µm thick brain slices as previously described in our laboratory [35]. Immunofluorescence was performed washing 3 times with 0.01 M PBS with 0.2% Triton X-100 (PBS-T). Subsequently, the slices were incubated in 0.15 M Glycine and 10 mg/mL NaBH4 to decrease background auto-fluorescence. Slices were rewashed with PBS and PBS-T and blocked with 3% BSA for 1.5 h at room temperature to avoid non-specific binding. After PBS and PBS-T washes, 30 µm thick brain slices were used for the detection of GFAP (Glial fibrillary acidic protein), Iba-1 (Ionized calcium-binding adaptor molecule), 8-OHdG (8-hydroxy-2’-deoxyguanosine), and 4-HNE (4-Hydroxynonenal) incubated overnight in PBS-T containing 0.5% BSA at 4 °C. To detect primary antibodies, sections were washed with PBS-T and incubated for 2 h at room temperature with the respective secondary antibody in PBS-T containing 0.5% BSA. Then, brain slices were washed with PBS-T, PBS, and water, mounted on gelatin-coated slides, and cover with a fluorescence mounting medium.

### 2.5. Image Analysis

The brain slices subjected to immunohistochemistry were photographed with an Olympus BX51 microscope coupled to an RTV 3.3 Micropublisher camera (QImaging, Surrey, BC, Canda). Photographs were analyzed using ImageJ 1.49.v (NIH) software. The measurement was performed by manual adjustment of the threshold or by manual selection of the ROI in all images. Quantifications were performed blindly in 9-12 coronal sections per animal. The relative fluorescence intensity was calculated as the intensity after subtraction of the background noise and the analysis of GFAP and Iba-1 somas was performed by the corrected total cell fluorescence (CTCF = Integrated Density−(Area of selected cell X Mean fluorescence of background readings). Three animals were used per experimental group. CTCF was measured, using 10 cells per image, 5 images per animal. The analysis of each picture was carried out by normalizing for the average intensity of WT Sham groups in each experiment.

### 2.6. Memory Flexibility Test

The memory flexibility (MF) test was performed as previously described [36]. The mice were trained in a circular water maze of 1.1 m in diameter, which contains opaque water at 19–21 °C, 50 cm deep, which has a platform of 9 cm in diameter, submerged 1 m below the level of the water. Each round of training has a maximum duration of 60 s, or until the platform is found. At the end of the round, the animal is left on the platform for 10 s. The time between rounds is 10 to 15 min. Each animal was trained to find the pseudo-random location of the platform for 4 days, with a new location each day of training. A maximum of 10 training trials was performed until the animal reached the exclusion criterion (3 successive trials with an escape latency of less than 20 s). After completion of the trial, the mice were returned to their cages. Data were collected using a water maze video tracking system (ANY-maze video tracking software, Stoelting Co, Wood Dale, IL, USA).

### 2.7. Novel Object Recognition Test

The New Object Recognition (NOR) task is based on the observation that rodents focus on novel objects, thus spending more time exploring these new objects as compared to objects already known to them. The new recognition test was carried out as previously described [37,38,39]. Before the test, the animal was subjected to a period of training to accustom it to the environment. This was done by leaving the animal in the center of the 60 × 60 cm acrylic box and allowing it to explore it freely for 10 min for three consecutive days. The next day, two identical objects were placed in the box, and the animal was allowed to explore the objects for 10 min (acquisition phase). 5–6 h after the acquisition phase, the animals were put back into the box, keeping a known object and a new object (recognition phase). The movement of the mice was recorded using a video tracking system (ANY-maze video tracking software, Stoelting Co, Wood Dale, IL, USA). The time spent exploring the new object about the time spent exploring the familiar object was used as a memory index. The box and objects were cleaned after each round to remove any olfactory signals. The objects used in these experiments were based on preliminary experiments to choose objects that did not have a predetermined bias based on their size, texture, color, or geometry.

### 2.8. Immunoblotting

The hippocampus of mice from all experimental groups was dissected on ice and immediately processed as previously described [19]. The mouse hippocampal samples were homogenized in RIPA buffer (10 mM Tris-Cl, pH 7.4, 5 mM EDTA, 1% NP-40, 1% sodium deoxycholate, and 1% SDS), supplemented with inhibitors of proteases (Amnesco, VWR Life Science, Suwanee, GA, USA) and phosphatases (25 mM NaF, 100 mM Na_3_VO_4_ and 30 µM Na_4_P_2_0_7_), using a Potter homogenizer. The samples were centrifuged twice at 14,000 rpm at 4 °C for 15 min. The protein concentration is determined using a BCA protein assay kit (Pierce). Samples were resolved by SDS-PAGE, followed by immunoblotting onto PVDF membranes. Immunodetection was performed with their respective primary antibodies incubated overnight at 4 °C followed by detection with secondary antibodies coupled to peroxidase and revealed using a chemiluminescence detection kit (West Pico, ThermoFisher, Waltham, MA, USA). The images were obtained with a G: BOX Chemi XT4 Gel Imaging system (Syngene, Bangalore, India).

### 2.9. Subcellular Fractionation

Subcellular fractionation was performed in hippocampal tissue from WT and SOD2^+/−^ mice after TBI using an adapted protocol [13]. Hippocampal samples were dissected into the cold artificial cerebrospinal fluid (ACSF; in mM: 124 NaCl, 2.6 NaHCO_3_, 10 D-glucose, 2.69 KCl, 1.25 KH_2_PO_4_, 2.5 CaCl2, 1.3 MgSO4, and 2.60 NaHPO_4_) and homogenized in 200 μL of buffer A (320 mM sucrose, 5 mM HEPES, pH 7.4) containing a ’‘complete’ protease inhibitor cocktail (Roche Diagnostics, Laval, QC., Canada). The samples were centrifuged for 10 min at 1000× *g* at 4 °C. The supernatant obtained (fraction S1) was centrifuged at 12,000× *g* for 20 min at 4 °C. This centrifugation resulted in the supernatant (fraction S2; microsomes and cytosol) and the sediment (fraction P2; crude synaptosomal membranes). The S2 fraction was centrifuged at 160,000× *g* for 2 h at 4 °C to separate the cytosolic (supernatant) and microsomal (pellet) fraction. The microsomal pellet was resuspended in 100 µL of buffer B (4 mM HEPES, 1 mM EDTA, pH 7.4). The P2 fraction was resuspended in buffer C (20 mM HEPES, 100 mM NaCl, 0.5% Triton, pH 7.2) and was left in slow rotation for 15 min at 4 °C, to then be centrifuged at 12,000× *g* for 20 min at 4 °C. The supernatant (NP fraction soluble in triton) containing the non-PSD membranes was stored. The pellet was resuspended in 120 μL of buffer D (20 mM HEPES, 0.15 mM NaCl, 1% Triton-X, 1% deoxycholic acid, 1% SDS, 1 mM DTT, pH 7.5), followed by a gentle rotation (1 h, 4 °C) and of centrifugation at 10,000× *g*, for 15 min at 4 °C. The pellet was discarded, and the supernatant was retained (insoluble PSD fraction of Triton X-100). Microsomal, cytosolic, PSD, and non-PSD samples were stored at −80 °C until use, as previously described [34].

### 2.10. Slice Preparation and Electrophysiology

For the electrophysiological recordings, slices of the dorsal hippocampus were cut transversely at 400 µm in cold ACSF. A vibratome (BSK microslicer DTK-1500E, Ted Pella, Redding, CA, USA) was used to cut the brain slices. The brain slices were incubated in ACSF for 1 h at room temperature [19,40]. The brain slices were transferred to an experimental chamber (2 mL), with a solution exchange of 3 mL/min, and 10 µM PTX, an inhibitory transmission inhibitor corresponding to GABA-A, was added. To evoke field excitatory postsynaptic potentials (fEPSPs), we stimulated with concentric bipolar electrodes (tungsten, 125 µm OD, microprobes) connected to an isolation unit (Isoflex, AMPI, Jerusalem, Israel). Stimulation was on the Stratum Radiatum within 100–200 µm of the recording site. The records were filtered at 2.0–3.0 kHz, and recorded at 4.0 kHz using an A/D converter (National Instrument) and stored with the WinLTP program [41,42]. Baseline excitatory synaptic transmission was measured using an input/output curve protocol consisting of 10 stimuli ranging from 200 to 900 μA (the interval between stimuli was 10 s). To generate LTP, we used high-frequency stimulation (HFS), which consisted of 2 trains at 100 Hz of stimuli with an interval between trains of 10 s. Data was collected and analyzed offline with pClamp 10 (Molecular Devices, San Jose, CA, USA).

### 2.11. Statistical Analysis

All results were analyzed with Prism (GraphPad Software Inc., San Diego, CA, USA) and are presented as mean ± standard error. The data were analyzed using one-way or two-way Anova, as appropriate, followed by the post, Bonferroni’s test. Values of *p* ≤ 0.05 were considered for statistically significant differences.

## 3. Results

### 3.1. TBI Generates Neuroinflammation and Oxidative Stress in WT and SOD2^+/−^ Animals

**Figure 1:**
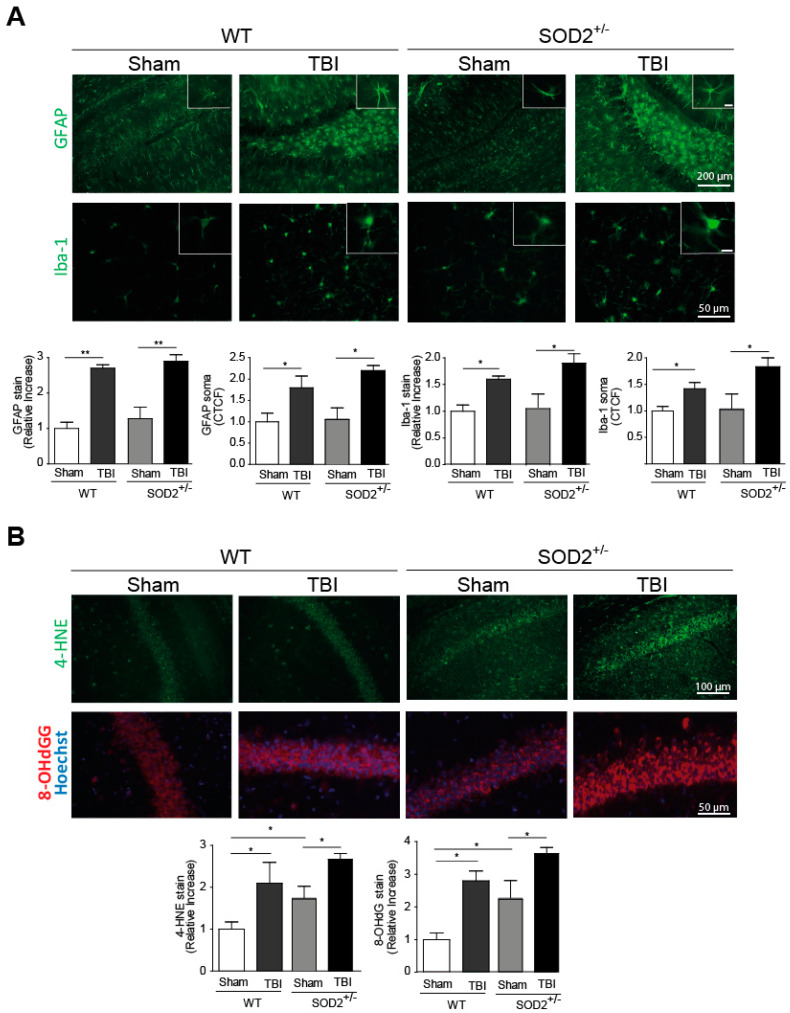
Neuroinflammation and oxidative stress are increased in hippocampal slices from mice after TBI. (**A**). Representative immunofluorescence images showing GFAP and Iba-1 in hippocampal slices from mice 1 week after TBI. The graphs show the quantification of fluorescence intensity in the selected area of hippocampi and CTCF of soma from GFAP and Iba-1 reactive cells. Inset scale bar correspond to 10 μm. (**B**). Representative immunofluorescence images showing 4-HNE and 8-OHdG in hippocampal slices from mice 1 week after TBI, specifically in the CA1 region. The graphs show the quantification of fluorescence intensity in the CA1 hippocampal region. 3 animals were used per experimental group and 9–12 images per animal were analyzed. CTCF was measured, using 10 cells per image, 5 images per animal. Data are means ± S.E and are relativized to the WT Sham group. Statistical differences were calculated by ANOVA, followed by post hoc Bonferroni’s test. Asterisks indicate the statistical significance of the observed differences (* *p* < 0.05; ** *p* < 0.01). (GFAP, Glial fibrillary acidic protein; TBI, Traumatic brain injury; Iba-1, Ionized calcium-binding adaptor molecule 1; 4-HNE, 4-Hydroxynonenal; 8-OHG: 8-hydroxy-2′-deoxyguanosine, CTCF: corrected total cell fluorescence).

Neuroinflammation and increased oxidative stress markers are among the secondary damages produced by brain trauma, and they are locally activated by-products of cellular damage (DAMPs, Damage-Associated Molecular Patterns), such as ATP [2]. To evaluate the presence of neuroinflammation, we evaluated two markers for this purpose, the fibrillar acid protein (GFAP), present in astrocytes, and the Iba-1 microglia protein (Figure 1A). These markers were analyzed one week after the last session of trauma induction. The GFAP mark in the hippocampi and the average size of its somas increase at a one-week post-knock session (Figure 1A). In the case of Iba-1, the intensity of the total mark in the analyzed area and the average size of soma increases a one-week strongly after the induction of trauma (Figure 1A). In both neuroinflammation markers, WT and SOD2^+/−^ animals present a similar increase in these markers. Another secondary lesion analyzed is the accumulation of oxidative stress markers. In this case, 4-HNE, a lipid peroxidation product, and 8-OHdG (Figure 1B), a product of oxidation of nitrogenous bases, were analyzed. In WT animals, 4-HNE and 8-OHdG levels present an increase one week after the last session of trauma induction. In the case of SOD2^+/−^ mice, the control group (Sham, 2-month-old animals) present high levels of these markers as we previously described [30], similar to the WT mice after TBI but increased strongly after the trauma induction (Figure 1B).

The model of mild/moderate brain trauma implemented generates important secondary lesions such as subarachnoid hemorrhage in the brain (Supplementary Figure S2B), neuroinflammation, and oxidative stress present in the hippocampus and without causing psychomotor damage in the animal (Supplementary Figure S2A), these controls in the model of trauma implemented to allow us to make a good comparison to the effects produced in mild/moderate trauma in with significant cognitive disabilities and long-term behavior alterations.

### 3.2. Behavioral Evaluation of WT and SOD2^+/−^ Mice Subjected to Brain Trauma

**Figure 2:**
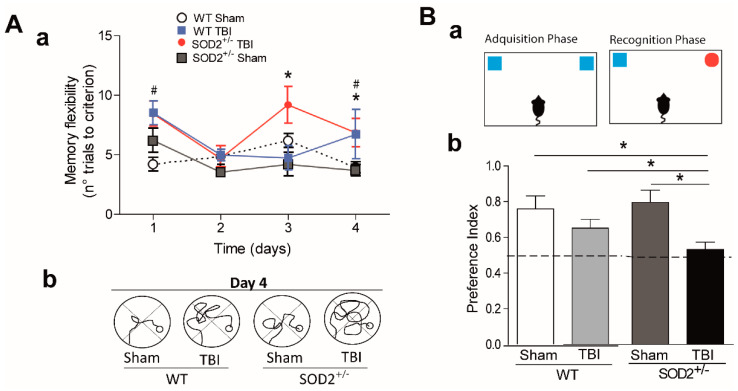
WT and SOD2^+/−^ mice show cognitive impairment after TBI. (**A**). Behavioral performance was evaluated by the memory flexibility test (**A**), and NOR test (**B**). Spatial memory of WT and SOD2^+/−^ mice after TBI was evaluated by the memory flexibility test (**Aa**). (**Ab**) Representative swimming trajectories for WT and SOD2^+/−^ mice of different conditions on day 4. An increase in the number of trials necessary to reach the criterion is observed in WT and SOD2^+/−^ sham animals versus WT and SOD2^+/−^ TBI animals. (**B**). TBI induces impairment performance in the NOR test in SOD2^+/−^ mice. 6 animals were used per experimental group. Data are means ± S.E. Statistically significant differences were calculated by one-way or two-way ANOVA and, followed by Bonferroni’s post hoc test. (Figure 2A: # *p* ˂ 0.5; between WT Sham and WT TBI; * *p* ˂ 0.5; between SOD2^+/−^ Sham and SOD2^+/−^ TBI; Figure 2B: * *p* ˂ 0.5; NOR, novel object recognition).

Cognitive deficit is the final consequence that occurs after brain trauma [8]. We proposed to intervene in the susceptibility to secondary damage in the animals submitted to the trauma protocol. We decided to decrease the antioxidant capacity in our trauma model using transgenic animals SOD2^+/−^ of 2 months of age. As we have previously observed, at two months of age, no cognitive changes were observed when we used a conventional configuration of the water maze [19]. We evaluated the cognitive performance of WT and SOD2^+/−^ mice subjected to brain trauma (Figure 2). For this purpose, we used a modified water maze test that allows evaluating spatial memory associated with episodic memory (memory flexibility, [43]), which is more sensitive in detecting hippocampal dysfunction [44] (Figure 2A). The analysis of behavioral performance indicates that WT animals subjected to trauma require more attempts to reach the learning criterion (the criterion corresponds to that the animals must find the platform in less than 20 s, three consecutive times, see Section 2.6 in methods) that the WT control animals in a couple of days of testing (Figure 2Aa,Ac). On the other hand, behavioral performance analysis indicates that SOD2^+/−^ animals subjected to trauma also require a greater number of attempts to reach the learning criterion (Figure 2Ab,Ac). A deficit in the number of trials required to reach the escape criterion was found at each of the four platform locations for the WT and SOD2^+/−^ TBI animals. As observed in Figure 2Aa, controls animals (WT and SOD2^+/−^ Sham) required a significantly reduced number of trials to reach the criterion (Figure 2Aa). When the strategies used by the animals to reach the platform were compared at day 4 (times <20 s), it was possible to appreciate the difference between Sham animals (WT and SOD2^+/−^) and TBI animals. TBI animals showed impaired strategies compared with controls (Figure 2Ab).

Another behavioral analysis used was the new object recognition test (NOR) (Figure 2B). This analysis takes advantage of the innate tendency of mice to explore a novel object more than a familiar one [37,38]. In the case of WT animals subjected to brain trauma, this test did not show significant differences in comparison to the control group (Figure 2B). In the case of animals SOD2^+/−^, the control group does not present alterations in the index of preference for the new object. In contrast, the SOD2^+/−^ animals subjected to brain trauma do not present a significant difference in the preference index, and they are significantly different from the control group for the novel object indicating a cognitive failure in this experimental group (Figure 2B). In summary, these results indicate that the low antioxidant capacity caused by the low expression of SOD2 is not enough to produce cognitive damage at two months of age. However, the accumulation of oxidative stress in SOD2 animals due to brain trauma could compromise the cognitive performance dependent on the hippocampus.

### 3.3. Evaluation of the Plasticity and Synaptic Response of WT and SOD2^+/^^−^ Mice Subjected to Brain Trauma

**Figure 3:**
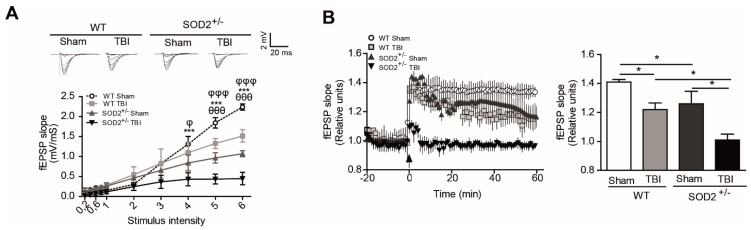
WT and SOD2^+/−^ mice show synaptic failure and synaptic plasticity impairment in the hippocampal circuit after TBI. (**A**). fEPSP slope induced by the input-output protocol to record total responses from WT and SOD2^+/−^ mice after TBI. (**B**). LTP was generated by HFS in the hippocampal CA1 area in slices from WT and SOD^+/−^ mice after TBI. Quantification of fEPSP slope 60 min after HFS. Data are means ± S.E. Three animals were used per experimental group. 3–4 hippocampal slices per animal were analyzed. Statistically significant differences were calculated by one-way or two-way ANOVA and, followed by Bonferroni’s post hoc test. Symbols indicate the statistical significance of the observed differences. (Figure 3A: θθθ *p* ˂ 0.001; between WT Sham and WT TBI; φ *p* ˂ 0.05, φφφ *p* ˂ 0.001 between SOD2^+/−^ Sham and SOD2^+/−^ TBI; *** *p* ˂ 0.001 between WT TBI and SOD2^+/−^ TBI. Figure 3B: * *p* ˂ 0.05). (LTP, Long- term potentiation; fEPSP, field excitatory postsynaptic potential).

To evaluate the effect of accumulation of oxidative stress on synaptic transmission after brain trauma, we have recorded synaptic activity in the collateral hippocampal circuit of Schaffer-CA1, measuring excitatory postsynaptic field evoked potentials (fEPSP) in WT and SOD2^+/−^ mice. We have performed input/output curve experiments to evaluate the synaptic strength on WT and SOD2^+/−^ mice after TBI (Figure 3A). We found a significant decrease in the total response to different stimulation intensities in WT mice subjected to brain trauma compared to WT control animals (Figure 3A). Regarding the 2-month-old SOD2^+/−^ used as control of the transgenic animals, we observed a decline in the total response compared to the WT control animals and an even stronger decrease in SOD2^+/−^ mice subjected to brain trauma (Figure 3A). Consistent with the idea that an increase in the cognitive deficit and certain markers of oxidative stress can compromise synaptic strength, we have evaluated the synaptic plasticity in WT and SOD2^+/−^ animals subjected to brain trauma (Figure 3B). We have found that LTP induction is compromised in WT mice subjected to brain trauma compared to control animals of the same age (Figure 3B). On the other hand, the 2-month-old SOD2^+/−^ animals presented a deficient LTP expression in comparison to a WT animal of the same age, while the SOD2^+/−^ animals subjected to the trauma induction were unable to generate potentiation (Figure 3B). These data strongly suggest that brain trauma alters the strength and synaptic plasticity, and these effects are more severe without the antioxidant barrier of the SOD2 enzyme.

**Figure 4:**
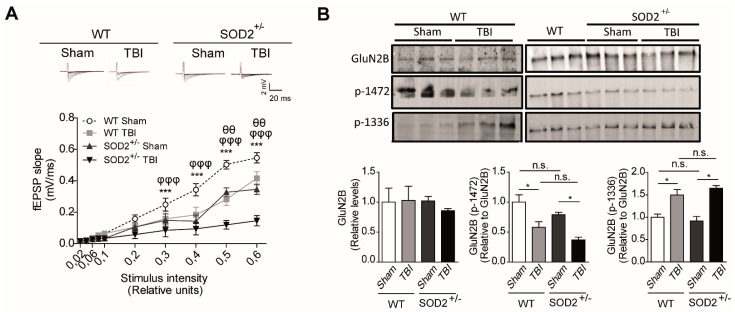
WT and SOD2^+/−^ mice showed failure in NMDA synaptic response and NMDA subunit phosphorylated status after TBI. (**A**). fEPSP slope induced by the input-output protocol to record responses corresponding to NMDARs from WT and SOD2^+/−^ mice after TBI. (**B**). Immunoblot analyses of phosphorylated GluN2B subunits of NMDARs and densitometric analysis of tyrosine 1472 and tyrosine 1336 phosphorylation in hippocampi lysates from WT and SOD2^+/−^ mice after TBI. Three animals were used per experimental group. 3–4 hippocampal slices per animal were analyzed Statistically significant differences were calculated by one-way or two-way ANOVA, followed by Bonferroni’s post hoc test. Symbols indicate the statistical significance of the observed differences. (Figure 4A: θθ *p* ˂ 0.01; between WT Sham and WT TBI; φφφ *p* ˂ 0.001 between SOD2^+/−^ Sham and SOD^2+/−^ TBI; *** *p* ˂ 0.001 between WT TBI and SOD^2+/−^ TBI. Figure 4B: * *p* ˂ 0.05, n.s.: non-significant).

Additionally, we analyzed in more detail the contribution of NMDARs within the synaptic response after the induction of brain trauma in WT and SOD2^+/−^ mice through electrophysiological and biochemical assays (Figure 4). We found a significant decrease in the synaptic response of the NMDARs (records made without Mg^+2^, with 20 μM of NBQX) at different intensities of stimulation in WT mice subjected to brain trauma compared to WT control mice (Figure 4A). Regarding the 2-month-old SOD2^+/−,^ we have observed a decrease in the total response compared to WT control animals and an even stronger decrease in the SOD2^+/−^ mice subjected to brain trauma. (Figure 4A). As we have found a decrease in the response corresponding to NMDARs, we decided to analyze changes in the distribution of NMDARs between synaptic and extrasynaptic regions that could contribute to the neuropathological effects observed after brain trauma in WT and SOD2^+/−^ mice (Figure 4B).

As previously explained, numerous reports have shown that certain phosphorylations in tyrosine residues in the GluN2B subunit of NMDARs are strongly associated with their expression on the surface. In summary, tyrosine 1472 phosphorylation of GluN2B subunit, which regulates the expression on the synaptic surface of NMDARs [22], and phosphorylation at tyrosine residues 1336, which is associated with the enrichment of NMDAR receptors on extrasynaptic membranes [23]. Biochemical analysis among the WT control animals and WT animals subjected to brain trauma, no significant differences were found between the total levels of the GluN2B subunit. However, we observed a 40% (42.1 ± 7.8) decrease in phosphorylation that keeps NMDARs in the synaptic zone (tyrosine 1472) in WT animals subjected to brain trauma compared to control animals. On the other hand, the phosphorylation that enriches NMDARs in the extrasynaptic zone (tyrosine 1336) increases approximately 60% (64.7 ± 5.1) in mice in animals subjected to trauma (Figure 4B). When analyzing the same parameters in the SOD2^+/−^ animals of 2 months of age, we have not observed any significant differences between the total levels of the GluN2B subunit. However, the phospho-tyrosine 1472 label decreases by 20% in the SOD2^+/−^ control animals and drops further by about 50% (49.7 ± 10.5) in the same animals subjected to brain trauma.

**Figure 5:**
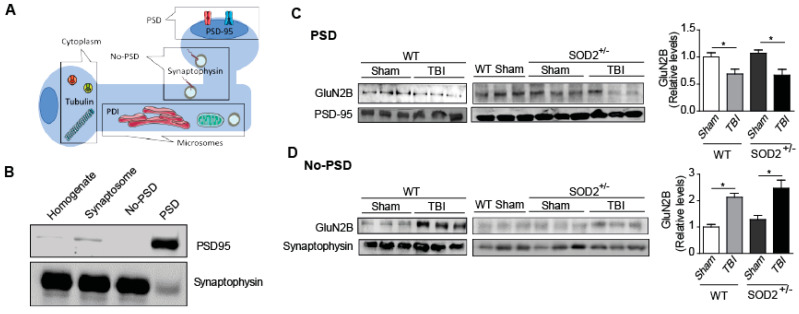
WT and SOD2^+/−^ mice showed alteration in NMDARs subunit distribution after TBI. Scheme (**A**) and characterization (**B**) of proteins in the cytoplasm, synaptic, extrasynaptic, and intracellular membrane fractions. Representative western blot with equal amounts of total protein loaded (20 µg) from each of the fraction probed with PSD marker PSD-95, the ER protein PDI, tubulin for cytoplasmatic fraction, and synaptophysin for the extrasynaptic fraction. Comparison of relative levels of NMDARs subunit Glun2B in synaptic (**C**) and extrasynaptic (**D**) fraction from hippocampi of WT and SOD2^+/−^ under TBI condition. Six animals were used per experimental group. Statistically significant differences were calculated by one-way ANOVA, followed by Bonferroni’s post hoc test. The asterisk indicates the statistical significance of the observed differences. (* *p* ˂0.05). (PSD, postsynaptic density).

On the other hand, the phospho-tyrosine 1336 labels of the animals SOD2^+/−^ control has relative levels similar to the WT animals, but the SOD2^+/−^ animals subjected to brain trauma present an increase of this phosphorylation close to 60% (Figure 4B). To confirm the effect of TBI on the distribution of NMDARs, which had only been analyzed through selective phosphorylations for each location, we made a subcellular fractionation protocol where, among several fractions, we were able to separate the postsynaptic zone from the extrasynaptic area (Figure 5). Within the markers used, we used synaptophysin, a protein associated with presynaptic fractions but also present in non-synaptic areas, and PSD-95, a scaffolding protein in the postsynaptic zone (Figure 5A,B) [13,34,45]. When analyzing the levels of the GluN2B subunit in the postsynaptic zone, we observed a decrease in the WT animals after TBI in comparison with the WT control animals (Figure 5C). In the case of the SOD2^+/−^ animals, we have not observed changes in comparison to WT control animals, but we observe strong decreases when SOD2^+/−^ subjected to the trauma protocol (Figure 5C). Analysis of the non-synaptic fraction reveals a strong increase of the GluN2B subunit in this fraction in the WT animals after the trauma induction session (Figure 5D). In the case of the SOD2^+/−^ animals, a strong increase was observed in SOD2^+/−^ animals subjected to brain trauma (Figure 5D).

These results suggest that brain trauma and low antioxidant barrier in animals can induce pathological changes in synaptic function of the hippocampus and affect hippocampal function, possibly in part due to the reduction of synaptic NMDARs and the increase in the population of extrasynaptic NMDARs.

### 3.4. The Signaling Associated with NMDARs Is Altered in WT and SOD2^+/−^ Mice Subjected to Brain Trauma

**Figure 6:**
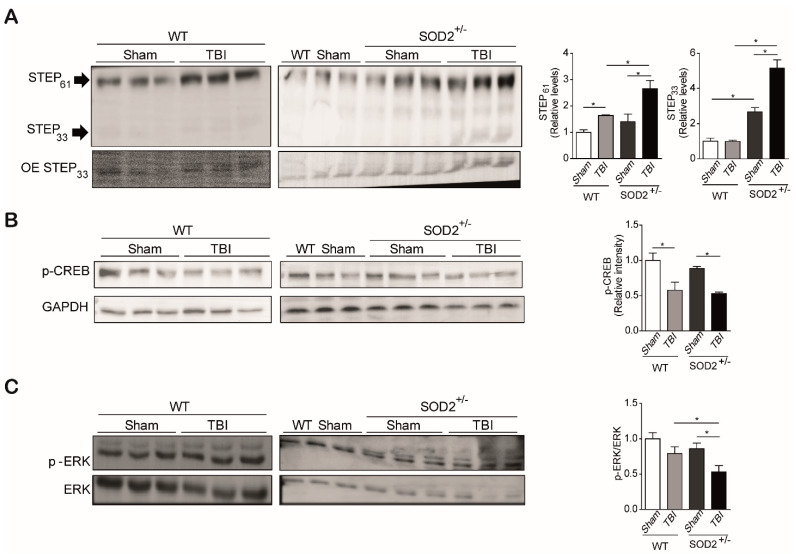
STEP_61_ and NMDARs associated signaling are altered in WT and SOD2^+/−^ mice after TBI. Immunoblot and densitometric analysis of STEP_61_, STEP_33_ (**A**), p-CREB (**B**), and p-ERK (**C**) from hippocampi lysates from WT and SOD2^+/−^ mice after TBI. Three animals were used per experimental group. Data are means ± S.E. Statistical differences were calculated by ANOVA, followed by post hoc Bonferroni’s test. Asterisks indicate the statistical significance of the observed differences (* *p* <0.05). OE, overexpression.

Because we observed an increase in the population of extrasynaptic NMDARs, detected by tyrosine phosphorylation 1336, and a reduction in the synaptic form of NMDARs, detected by tyrosine phosphorylation in 1472 in WT and SOD2^+/−^ animals subjected to trauma cerebral, we have analyzed several related targets in the signaling related to the NMDARs. These targets are CREB, STEP_61_, and ERK (Figure 6). As previously mentioned, the synaptic activity triggered by the activation of NMDARs induces gene transcription mediated by CREB. CREB translocates to the nucleus by phosphorylation at the serine 133 residues, and it is used as a marker of transcriptional activity. It has also been reported that this brand of transcriptional activity decreases with the activation of extrasynaptic NMDARs [12,15,46].

The analysis of p-CREB (S133) in the hippocampus of WT and SOD2^+/−^ mice was performed by immunoblot assays. It could be observed that cerebral trauma decreases p-CREB levels, both in WT animals and SOD2^+/−^ animals (Figure 6B). These data are in agreement with the decrease of the synaptic signaling mediated by the NMDARs observed previously (Figure 4). On the other hand, we analyzed a phosphatase that directly and indirectly is involved in the phosphorylation state of the NMDARs, STEP_61_. As explained above, this phosphatase regulates the phosphorylation state through 2 pathways, the direct dephosphorylation of tyrosine 1472 or indirectly through the dephosphorylation and inactivation of the Fyn kinase, responsible for this phosphorylation [25,47]. The analysis of the WT animals subjected to brain trauma showed an increase of over 50% in their expression level (Figure 6A). In the case of SOD2^+/−^ animals, no significant changes were observed between animals without trauma induction with WT animals. On the contrary, the group of SOD2^+/−^ animals subjected to brain trauma have an increase of more than 100% in their expression levels (Figure 6A). In addition to measuring the total levels of STEP_61_, its activity was measured by another target of this phosphatase, which is the phosphorylated version of ERK [48]. In the case of WT control animals and subjected to trauma, no significant changes were observed in phosphorylated ERK levels (Figure 6C). In contrast, the SOD2^+/−^ animals show a drastic decrease in p-ERK, suggesting that in addition to an increase in STEP_61_ levels, there is also a strong increase in their activity (Figure 6C). A similar increase in STEP_61_ levels has been reported for AD models and HD transgenic mice models [17,29]. STEP_61_ is the active form of this phosphatase, while STEP_33_ is the degradation product associated with the activation of calpains by the stimulation of extrasynaptic NMDARs [17,49,50]. No significant differences were observed in the STEP_33_ levels in the WT control animals, and those subjected to brain trauma, this version of STEP_61_ is found at such low levels that it was necessary to overexpose the membrane at the time of revealing (Figure 6A). In the case of SOD2^+/−^ animals subjected to brain trauma, the presence of STEP_33_ increases several times compares to control animals, showing that in addition to the increase in the population of the extrasynaptic NMDARs, there is also an activation of this receptor population.

These results indicate that the damages produced in the hippocampus after brain trauma alter various signaling pathways, among which are the pathways that mediate the distribution of NMDARs, in this case demonstrating an imbalance of a phosphatase involved in the phosphorylation state of NMDARs, which have also been altered in several other neuropathologies that could be mediating the neuropathological events present in this model of brain trauma.

### 3.5. The Pharmacological Inhibition of STEP_61_ Restores the Damage Produced after Brain Trauma

The previously observed results showed deregulation in the phosphorylation states of the GluN2B subunit of the NMDARs, which influences its location at the neuronal level and its functionality (Figure 6) [23]. This change in the phosphorylation states of the GluN2B subunit after brain trauma occurs associated with an increment in STEP_61_ phosphatase levels (Figure 6). This phenomenon has been observed in other neuropathological conditions as AD, HD and as a result of aging and the advanced redox stage [17,19,29,51]. For this reason, we decided to administer a pharmacological inhibitor for STEP_61_ phosphatase to reverse the effects produced after brain trauma attributed to changes in phosphorylation of NMDARs. The pharmacological inhibition of STEP_61_ was performed using the compound TC-2153 (benzopentathiepin 8-(trifluoromethyl)-1,2,3,4,5-benzopentathiepin-6-amine hydrochloride), belonging to a new family of tyrosine phosphatase inhibitors based on cyclic polysulfurized pharmacophores that form reversible covalent bonds with the catalytic cysteine in STEP_61_ [29]. This inhibitor proved to have a high specificity in the inhibition of STEP_61_ compared to other phosphatases; besides, no toxicity phenomena were observed in neuronal cultures, and it is capable of reversing the cognitive deficit in the AD mice model without affecting the classical markers of the disease (Tau phosphorylation and β-amyloid deposits) [29].

**Figure 7:**
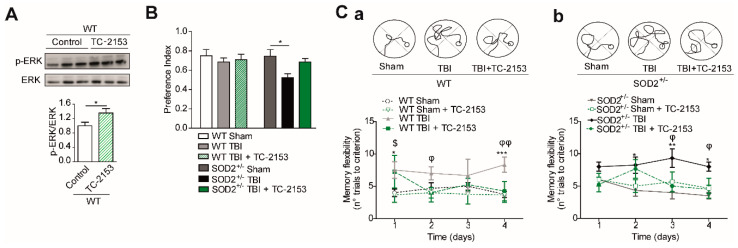
Pharmacological inhibition of STEP_61_ restores the cognitive impairment induces by TBI in WT and SOD2^+/−^ mice. (**A**). Pharmacological inhibition of STEP_61_ by intraperitoneal injection of TC-2153 10 µg/Kg measured by p-ERK level in the western blot analysis. (**B**). NOR test of WT and SOD2^+/−^ under TBI condition and treated with TC-2153. (**C**). Spatial memory of WT (**Ca**) and SOD2^+/−^ (**Cb**) mice after TBI and treated with TC-2153 was evaluated by the memory flexibility test. Three animals were used per experimental group for western blot analysis, and animals were used per experimental group for NOR and MF tests. Data are means ± S.E. Statistically, significant differences were calculated by one-way or two-way ANOVA, followed by post hoc Bonferroni’s test. Symbols indicate the statistical significance of the observed differences (Figure 7A,B: * *p* < 0.05; Figure 7Ca: * *p* ˂ 0.05, *** *p* ˂ 0.001 between WT Sham and WT TBI, $ *p* ˂ 0.01 between WT Sham and WT TBI + TC-2153, φ *p* ˂ 0.05, φφ *p* ˂ 0.01 between WT TBI and WT TBI + TC-2153; Figure 7Cb, * *p* ˂ 0.05, ** *p* ˂ 0.01 between SOD2^+/−^ Sham and SOD2^+/−^ TBI; φ *p* ˂ 0.05 between SOD2^+/−^ TBI and SOD2^+/−^ TBI + TC-2153).

**Figure 8:**
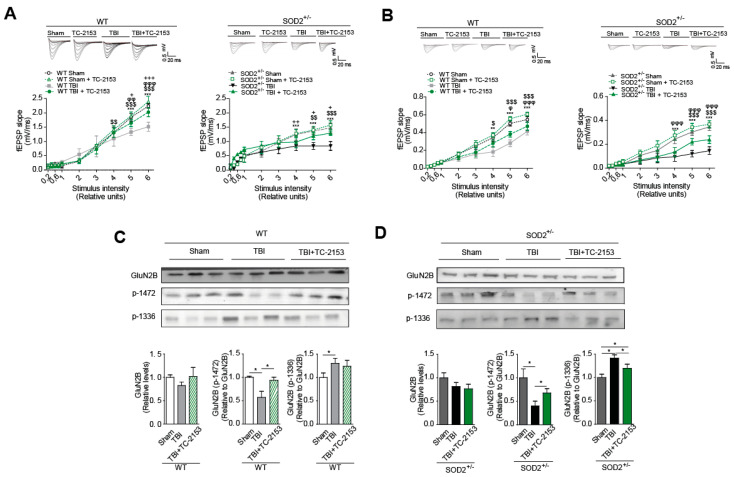
Pharmacological inhibition of STEP_61_ restores the NMDA synaptic response and NMDA subunit phosphorylated status after TBI. fEPSP slope induced by the input-output protocol to record total responses (**A**) and responses corresponding to NMDARs (**B**) from WT and SOD2^+/−^ mice after TBI. C. Immunoblot analyses of phosphorylated GluN2B subunits of NMDARs and densitometric analysis of tyrosine 1472 and tyrosine 1336 phosphorylation in hippocampi lysates from WT (**C**) and SOD2^+/−^ (**D**) mice after TBI. Three animals were used per experimental group. 3–4 hippocampal slices per animal were analyzed. Statistically significant differences were calculated by one-way or two-way ANOVA, followed by Bonferroni’s post hoc test. Symbols indicate the statistical significance of the observed differences. (Figure 8A,B for WT mice: *** *p* ˂ 0.001; between WT Sham and WT TBI, $ *p* ˂ 0.05, $$ *p* ˂ 0.01, $$$ *p* ˂ 0.001 between WT Sham + TC-2153 and WT TBI + TC-2153, φ *p* ˂ 0.05, φφ *p* ˂ 0.01, φφφ *p* ˂ 0.001 between WT TBI and WT TBI + TC-2153; ++ *p* ˂ 0.01, +++ *p* ˂ 0.001 between WT Sham and WT TBI + TC-2153; Figure 8A,B for SOD2^+/−^ mice: * *p* ˂ 0.05, ** *p* ˂ 0.01, *** *p* ˂ 0.001 between SOD2^+/−^ Sham and SOD2^+/−^ TBI; ++ *p* ˂ 0.01, +++ *p* ˂ 0.001 between SOD2^+/−^ Sham and SOD2^+/−^ TBI + TC-2153; $$ *p* ˂ 0.01, $$$ *p* ˂ 0.001 between SOD2^+/−^ Sham + TC-2153 and SOD2^+/−^ TBI + TC-2153; φ *p* ˂ 0.05, φφφ *p* ˂ 0.001 between SOD2^+/−^ TBI y SOD2^+/−^ TBI + TC-2153; Figure 8C,D: * *p* < 0.05).

STEP_61_ inhibitor TC-2153 was administered at a dose of 10 mg/Kg of animal weight (See materials and methods and Supplementary Figure S1). In this case, if the effectiveness to reverse changes produced after the induction of brain trauma was demonstrated. The dose used had already been previously reported [29,51], but in any case, we evaluated the effectiveness of the treatment through a known target of STEP_61_ phosphatase activity, which is the phosphorylation of ERK [52,53]. Treatment with TC-2153 proved effective as an inhibitor, achieving an increase in p-ERK compared to the control group treated with saline (Figure 7A). Cognitive performance analysis revealed an improvement after treatment with TC-2153 evaluated by the new object recognition test and the memory flexibility test (Figure 8B,C, respectively). In the memory flexibility test case, an improvement was observed in WT animals subjected to trauma treated with TC-2153 compared to those treated with saline (Figure 7Ca). In the case of SOD2^+/−^ animals, the reversal of damage induced by brain trauma after treatment with TC-2153 is even more evident (Figure 7Cb). Previously, it had been shown that brain trauma induces changes in total synaptic responses and corresponding responses to NMDARs (Figure 5 and Figure 6).

For this reason, it was evaluated whether the pharmacological inhibition of STEP_61_ restores the changes produced by brain trauma. Indeed, treatment with TC-2153 restores synaptic responses in both WT and SOD2^+/−^ animals (Figure 8A). A similar trend was observed when evaluating the currents corresponding to the NMDARs; TC-2153 treatment restored the loss of response after the induction of cerebral trauma in both WT and SOD2^+/−^ animals (Figure 8B). As previously explained, numerous reports have shown that certain phosphorylations in tyrosine residues in the GluN2B subunit of NMDARs are strongly associated with their expression on the surface and that this balance in the phosphorylation state of the NMDARs and therefore of its location at the neuron level is altered after the induction of brain trauma [23] (Figure 8C,D). Phosphorylation in tyrosine 1472, which regulates the expression on the synaptic surface of NMDARs [22], decreases significantly after brain trauma (Figure 5 and Figure 7). In WT animals, treatment with TC-2153 managed to restore part of the decrease observed after brain trauma (Figure 8C). In the case of the SOD2^+/−^ animals, the restoration of the tyrosine phosphorylation levels 1472 of the NMDARs is even more evident (Figure 8D). In the case of phosphorylation in residues of tyrosine 1336, which is associated with the enrichment of NMDARs in extrasynaptic membranes [23], an increase in this phosphorylation after the brain trauma was previously observed (Figure 4). After treatment with TC-2153, levels of p-1336 showed no significant changes in WT animals (Figure 8C). The opposite occurred with the SOD2^+/−^ animals, where there was a significant decrease in this phosphorylation after treatment with TC-2153 (Figure 8D).

These data strongly suggest the key role of STEP_61_ phosphatase in the mechanism of damage generated after brain trauma that includes alterations in the state of phosphorylation of NMDARs and that its pharmacological inhibition helps reverse the observed changes at the cognitive and molecular level.

## 4. Discussion

Using a transgenic model with compromised antioxidant capacity (low activity of the SOD2 enzyme), we previously demonstrated that, under conditions of oxidative stress, there is deregulation in intracellular signaling associated with the distribution and/or location of NMDARs, which triggers changes in the hippocampus-dependent functions with consequences on the memory and learning levels [19]. Here, we have demonstrated the susceptibility that exists when presenting this decrease in the antioxidant capacity to damage under a model of mild traumatic brain damage, which causes these animals to present the characteristics of the animal in advanced age, in agreement with the previous data, with a high level of ROS markers at the tissue level [19]. There are mainly two mechanisms through which ROS affects ion channels: 1) by direct oxidation of amino acid residues or 2) interfering with the signaling pathways that regulate these channels [54,55,56]. The activity of NMDARs can be regulated by directly linked hydroxyl radicals at redox modulating sites of this receptor [54,57,58]. However, these receptors are not the only channels that are affected by ROS in the hippocampus affecting processes dependent on this structure, such as learning and memory. Ryanodine receptors (RyRs) are a family of channels that release calcium from intracellular organelles. The dysfunction of these channels sensitive to the oxidation state of their amino acids can generate alterations in the ability to generate LTP in some neuronal circuits [56].

In this work, we have focused on assessing how oxidative damage, generated through external damage (brain trauma), alter the signaling involved in the distribution of the NMDARs. Our hippocampal experiments of SOD2^+/−^ animals, which have a considerable decrease in the activity of SOD2 in the brain [31], show that oxidative stress affects the synaptic transmission mediated by NMDARs; this is especially drastic under trauma conditions. Furthermore, given the importance of NMDARs in synaptic transmission [9,59], deregulation of downstream signaling through oxidative damage provides a mechanism of synaptic dysfunction in several other neuropathological conditions where the increase of ROS has a fundamental regulatory role [60,61,62,63].

NMDARs are located in different compartments of the neuronal membrane, and all of them can activate different signaling pathways when activated by glutamate. For example, the activation of synaptic NMDARs is coupled with the activation of ERK, with the subsequent activation of CREB, signaling involved in synaptic strength and neuronal survival [15,64]. Also, previous reports indicate that the regulation of the GluN2B subunit by Cdk5 improves memory in rat models and that its increase in activity is associated with various types of brain damage [65,66,67,68]. In contrast, the activation of extrasynaptic NMDARs is associated with the activation of calpains, p38, and several other cell death pathways, together with the inhibition of the CREB pathway [11,12,46,69]. NMDARs are more dynamic than originally thought, and neurons can regulate the number, distribution, and composition of the subunit of synaptic and extrasynaptic receptors [11,20,70,71,72]. However, the mechanism and signals that control the presence of NMDARs in different cell domains are mainly dependent on the phosphorylation state of the GluN2B subunit [23]. Within the most important phosphorylation are two carried out in tyrosine residues.

Phosphorylation in tyrosine 1472 of the GluN2B subunit is critical to keep the NMDA receptor at the synapse and preventing its endocytosis [9,59,73]. On the other hand, phosphorylation in tyrosine 1336 of the GluN2B subunit is associated with an enrichment of extrasynaptic NMDARs [16,23]. Both phosphorylations are substrates of the kinase of the Src family called Fyn [22,74]. Phosphatases can also control the phosphorylation status of NMDARs, one of which is STEP_61_, which modulates the phosphorylation state through two parallel pathways: by direct dephosphorylation of the tyrosine residue of the GluN2B 1472 subunit [23,75], and indirectly via dephosphorylation and inactivation of the Fyn kinase [25]. Also, STEP_61_ phosphatase is differentially regulated by synaptic and extrasynaptic NMDARs. Under conditions of synaptic activity, STEP_61_ is ubiquitinated and degraded via the proteasome, eliminating it from the synaptic area, and, conversely, the decrease in synaptic stimuli contributes to the increase of this phosphatase [26,47,53,76]. On the other hand, the extrasynaptic stimulation of NMDARs activates calpains that hydrolyze STEP_61_, leaving the 33 KDa isoform as a degradation by-product (STEP_33_) [49]. The activity of STEP_61_ is regulated by phosphorylations. Phosphorylation in serine 221, performed by PKA, forms the inactive form of STEP_61_, while its dephosphorylated form, performed by protein phosphatase 1 (PP1), is the active form. Therefore, it is evident that the increase in STEP_61_ activity is increased by the decrease in STEP_61_ neuronal activity [49,75]. Also, this phosphatase under conditions of oxidative damage, as enhanced in neurons in culture using hydrogen peroxide, has demonstrated aggregation of this protein. This aggregation prevents its ubiquitination and degradation, thereby increasing its levels. As for their activity, small oligomers increase their activity, while with the more complex oligomers, a loss of their activity has been observed [30]. This oligomerization mechanism, together with the activation of STEP_61_ phosphatase modulated through synaptic activity, are key in the deregulation of STEP_61_ in various pathologies.

In the last few years, a great effort has been devoted to understanding how the deregulation of the phosphatase STEP_61_ contributes to the pathophysiology of various neuropsychiatric disorders such as AD, Huntington’s disease, schizophrenia, X-fragile syndrome, and in the pathological changes associated with aging [17,28,29,49,51,77,78]. Based on these antecedents and other epidemiological studies, the damage produced after brain trauma has been considered a chronic pathophysiological event with several markers and signs common with other neuropathological diseases [1,2]. Among the markers and signs in common with other neuropathologies are states of dementia, psychotic symptoms, depression, and a strong incidence of other pathologies that propose brain trauma as a risk factor [5,79]. In addition, abnormal protein aggregates such as those found in AD (neurofibrillary tangles and amyloid deposits) have been found at the model level [80,81,82,83], failure in the protein degradation system [84], neuroinflammation and oxidative damage in the hippocampus of these animals [85,86,87,88], an area of strong interest because memory and learning to depend on this area which is one of the most affected after these types of neuropathologies [89,90].

As for animal models of brain trauma, there is a wide variety of these, including open traumatic trauma, loss of cortical mass, and closed traumas caused by waves of expansion [91], cortical impacts, and frontal impacts [7,32]. Despite the existence of a wide variety of models, most of them have been limited to descriptive reports of different types of damage such as structural damage [92], cognitive and synaptic damage, and neuroinflammation [6,8,85,86,87,88], but few studies have demonstrated a possible mechanism of the long/medium-term damage produced after brain trauma. For this reason, we have decided to use a model of frontal trauma, which does not generate tissue or structural damage, to study the effects on the hippocampus and its functional implications, having as a study target the modulation of synaptic receptors through various signaling pathways, having the oxidative stress as a sign in common with other pathologies. In order to elucidate a possible mechanism of damage, a combination of biochemical, histological, electrophysiological, and behavioral tools has been used to identified downstream signaling to the activation of NMDARs and regulate their location in synaptic or extrasynaptic sites under different damage conditions. First, we have shown some harmful effects, such as oxidative stress, affecting the distribution of NMDARs, and the signaling that leads to this phenomenon. Initially, we begin by evaluating the effect of ROS generation on aging in WT mice and with reduced antioxidant capacity due to the low expression of the SOD2 enzyme. This model shows an increase in oxidative damage markers in WT mice at 6 months of age. As for SOD2^+/−^ animals, the levels of oxidative damage markers are elevated, the increase being more significant at 6 months of age. However, cognitive damage is only observable at 6 months of age, suggesting that this damage is caused by long exposure and the cumulative damage of ROS. As for the animals submitted to the brain trauma model, young animals, 2 months old, were used, age in which SOD2^+/−^ animals still do not show alterations [19].

In animals subjected to trauma, there is the generation of ROS and neuroinflammation, both phenomena present in the hippocampus, where we have also been able to observe cognitive and synaptic failure. Some of these effects observed after trauma are similar or sometimes more severe than those observed in older animals (6 months) [19]. Regarding alterations in synaptic transmission, mainly those related to NMDARs, present to SOD2^+/−^ mice of 6 months or to animals submitted to the trauma model may have their origin in a decrease in the synaptic form of NMDARs (measured by tyrosine phosphorylation 1472 of the GluN2B subunit) and/or increase in the population of extrasynaptic NMDARs (measured by tyrosine phosphorylation 1336 of the GluN2B subunit). The increase in this population can be triggered by STEP_61_ phosphatases as a result of the inactivation of the protein degradation system via proteasome and contribute to the decrease of the synaptic form of NMDARs [17,26,93,94]. The low activity related to NMDARs generated a decrease in the levels of phosphorylation in CREB, the target of the ERK pathway, reducing neuronal survival [15,19,46]. In our case, we have found a drastic reduction in p-CREB levels in animals subjected to brain trauma, demonstrating that downstream signaling to the activation of NMDARs is altered. Various neuropathological disorders such as AD, Parkinson’s disease, and HD show dysfunction in the signaling below the NMDARs receptors and deregulation of the oxidative state, suggesting that a common event, such as oxidative stress, may affect synaptic maintenance and its function [9,95]. The role of the oxidative state in the control of the distribution of NMDARs and downstream signaling offers a mechanism that provides a better understanding of the neuropathologies in which glutamatergic transmission is compromised. This mechanism described is one of the main contributors to the intracellular alterations of the neuron. We have focused on one effector of NMDAR signaling (STEP_61_) as possible intervention targets since the various neuronal survival, or death signaling pathways depend largely on the location of synaptically or extrasynaptically activated receptors. It should be noted that STEP_61_ phosphatase activity also affects AMPARs, an activity that also regulates their synaptic function in homeostatic synapse models and neuropathological events [49,96], so it increases its importance as a therapeutic target for different neuropathological conditions.

Neurons are particularly sensitive to oxidative damage due mainly to their inability to divide [55,97]. For this reason, the battery of antioxidant mechanisms is wide and diverse [62], and the malfunction of these components can become catastrophic for neurons. For example, in the neuropathological conditions that we have mentioned, in addition to oxidative stress and synaptic failure, there are alterations in other intracellular machinery such as alterations in the degradation and autophagy system [84,93,98,99], bioenergetic and mitochondrial alterations [100,101,102], neuroinflammation [85,103], and formation of protein aggregates [104,105]. In addition to the regulations studied here, including the increase in STEP_61_ activity, phosphatase than regulated the synaptic activity, which establishes a neuropathological mechanism not yet described for damage caused by general trauma, but if it has been previously described for other diseases such as AD, HD, and schizophrenia [17,28,49], what which reaffirms the concept that brain trauma should be treated as a chronic process rather than an acute phenomenon [2,8].

Understanding how glutamate receptors are regulated under conditions of oxidative stress-induced, and how this can be regulated by the presence of TBI, in various ways provides the basis for understanding the regulation of processes such as synapse formation, memory learning, and understanding various neuropathologies, opening opportunities for the development of treatments for diseases in which the distribution and signaling of NMDARs are compromised.

## 5. Conclusions

Oxidative stress and synaptic damage are two very common physiological processes in various neurodegenerative diseases. In this work, we have established the role of STEP_61_ phosphatase regulating the state of phosphorylation and the synaptic/extrasynaptic location of NMDARs under a model of acute oxidative stress, assessed through a moderate brain trauma model. Dysregulation of this phosphatase was observed, which contributed to the increase of the extrasynaptic population of NMDARs, which are responsible for much of the neuronal deterioration. These data suggest an important role for STEP_61_ in neurodegeneration caused by brain trauma, which could be used as a biomarker or pharmacological target.

## Data Availability

All data supporting the conclusions of this article are included within the article and supplementary materials.

## Supplementary Materials

The following are available online at https://www.mdpi.com/article/10.3390/antiox10101575/s1, Figure S1: Experimental design of mild traumatic brain injury model. Figure S2. Neurologic features after TBI induction. Table S1. List of antibodies used for western-blot analysis and immunofluorescence studies. Table S2: Neurological function scoring system.

## References

[1] Mollayeva T., Mollayeva S., Colantonio A. (2018). Traumatic brain injury: Sex, gender and intersecting vulnerabilities. Nat. Rev. Neurol..

[2] Davis A.E. (2000). Mechanisms of traumatic brain injury: Biomechanical, structural and cellular considerations. Crit. Care Nurs. Q..

[3] Lee S.M., Wong M.D., Samii A., Hovda D.A. (1999). Evidence for energy failure following irreversible traumatic brain injury. Ann. N. Y. Acad. Sci..

[4] Masel B.E., DeWitt D.S. (2010). Traumatic brain injury: A disease process, not an event. J. Neurotrauma.

[5] Jordan B.D. (2014). Chronic traumatic encephalopathy and other long-term sequelae. Continuum.

[6] Chen Y., Constantini S., Trembovler V., Weinstock M., Shohami E. (1996). An experimental model of closed head injury in mice: Pathophysiology, histopathology, and cognitive deficits. J. Neurotrauma.

[7] Xiong Y., Mahmood A., Chopp M. (2013). Animal models of traumatic brain injury. Nat. Rev. Neurosci..

[8] Davis A.E. (2000). Cognitive impairments following traumatic brain injury. Etiologies and interventions. Crit. Care Nurs. Clin. N. Am..

[9] Carvajal F.J., Mattison H.A., Cerpa W. (2016). Role of NMDA Receptor-Mediated Glutamatergic Signaling in Chronic and Acute Neuropathologies. Neural Plast..

[10] Mira R.G., Cerpa W. (2020). Building a Bridge between NMDAR-Mediated Excitotoxicity and Mitochondrial Dysfunction in Chronic and Acute Diseases. Cell. Mol. Neurobiol..

[11] Hardingham G.E., Bading H. (2010). Synaptic versus extrasynaptic NMDA receptor signalling: Implications for neurodegenerative disorders. Nat. Rev. Neurosci..

[12] Hardingham G.E., Fukunaga Y., Bading H. (2002). Extrasynaptic NMDARs oppose synaptic NMDARs by triggering CREB shut-off and cell death pathways. Nat. Neurosci..

[13] Milnerwood A.J., Gladding C.M., Pouladi M.A., Kaufman A.M., Hines R.M., Boyd J.D., Ko R.W., Vasuta O.C., Graham R.K., Hayden M.R. (2010). Early increase in extrasynaptic NMDA receptor signaling and expression contributes to phenotype onset in Huntington’s disease mice. Neuron.

[14] Wang Y., Briz V., Chishti A., Bi X., Baudry M. (2013). Distinct roles for mu-calpain and m-calpain in synaptic NMDAR-mediated neuroprotection and extrasynaptic NMDAR-mediated neurodegeneration. J. Neurosci. Off. J. Soc. Neurosci..

[15] Hardingham G.E., Arnold F.J., Bading H. (2001). Nuclear calcium signaling controls CREB-mediated gene expression triggered by synaptic activity. Nat. Neurosci..

[16] Gladding C.M., Raymond L.A. (2011). Mechanisms underlying NMDA receptor synaptic/extrasynaptic distribution and function. Mol. Cell. Neurosci..

[17] Gladding C.M., Sepers M.D., Xu J., Zhang L.Y., Milnerwood A.J., Lombroso P.J., Raymond L.A. (2012). Calpain and STriatal-Enriched protein tyrosine phosphatase (STEP) activation contribute to extrasynaptic NMDA receptor localization in a Huntington’s disease mouse model. Hum. Mol. Genet..

[18] Ivanov A., Pellegrino C., Rama S., Dumalska I., Salyha Y., Ben-Ari Y., Medina I. (2006). Opposing role of synaptic and extrasynaptic NMDA receptors in regulation of the extracellular signal-regulated kinases (ERK) activity in cultured rat hippocampal neurons. J. Physiol..

[19] Carvajal F.J., Mira R.G., Rovegno M., Minniti A.N., Cerpa W. (2018). Age-related NMDA signaling alterations in SOD2 deficient mice. Biochim. Biophys. Acta Mol. Basis Dis..

[20] Groc L., Bard L., Choquet D. (2009). Surface trafficking of N-methyl-D-aspartate receptors: Physiological and pathological perspectives. Neuroscience.

[21] Lau C.G., Zukin R.S. (2007). NMDA receptor trafficking in synaptic plasticity and neuropsychiatric disorders. Nat. Rev. Neurosci..

[22] Xu F., Plummer M.R., Len G.W., Nakazawa T., Yamamoto T., Black I.B., Wu K. (2006). Brain-derived neurotrophic factor rapidly increases NMDA receptor channel activity through Fyn-mediated phosphorylation. Brain Res..

[23] Goebel-Goody S.M., Davies K.D., Alvestad Linger R.M., Freund R.K., Browning M.D. (2009). Phospho-regulation of synaptic and extrasynaptic N-methyl-d-aspartate receptors in adult hippocampal slices. Neuroscience.

[24] Roche K.W., Standley S., McCallum J., Dune Ly C., Ehlers M.D., Wenthold R.J. (2001). Molecular determinants of NMDA receptor internalization. Nat. Neurosci..

[25] Nguyen T.H., Liu J., Lombroso P.J. (2002). Striatal enriched phosphatase 61 dephosphorylates Fyn at phosphotyrosine 420. J. Biol. Chem..

[26] Xu J., Kurup P., Zhang Y., Goebel-Goody S.M., Wu P.H., Hawasli A.H., Baum M.L., Bibb J.A., Lombroso P.J. (2009). Extrasynaptic NMDA receptors couple preferentially to excitotoxicity via calpain-mediated cleavage of STEP. J. Neurosci. Off. J. Soc. Neurosci..

[27] Basen-Engquist K., Coyle K.K., Parcel G.S., Kirby D., Banspach S.W., Carvajal S.C., Baumler E. (2001). Schoolwide effects of a multicomponent HIV, STD, and pregnancy prevention program for high school students. Health Educ. Behav. Off. Publ. Soc. Public Health Educ..

[28] Carty N.C., Xu J., Kurup P., Brouillette J., Goebel-Goody S.M., Austin D.R., Yuan P., Chen G., Correa P.R., Haroutunian V. (2012). The tyrosine phosphatase STEP: Implications in schizophrenia and the molecular mechanism underlying antipsychotic medications. Transl. Psychiatry.

[29] Xu J., Chatterjee M., Baguley T.D., Brouillette J., Kurup P., Ghosh D., Kanyo J., Zhang Y., Seyb K., Ononenyi C. (2014). Inhibitor of the tyrosine phosphatase STEP reverses cognitive deficits in a mouse model of Alzheimer’s disease. PLoS Biol..

[30] Deb I., Poddar R., Paul S. (2011). Oxidative stress-induced oligomerization inhibits the activity of the non-receptor tyrosine phosphatase STEP61. J. Neurochem..

[31] Van Remmen H., Salvador C., Yang H., Huang T.T., Epstein C.J., Richardson A. (1999). Characterization of the antioxidant status of the heterozygous manganese superoxide dismutase knockout mouse. Arch. Biochem. Biophys..

[32] Kilbourne M., Kuehn R., Tosun C., Caridi J., Keledjian K., Bochicchio G., Scalea T., Gerzanich V., Simard J.M. (2009). Novel model of frontal impact closed head injury in the rat. J. Neurotrauma.

[33] Mira R.G., Lira M., Quintanilla R.A., Cerpa W. (2020). Alcohol consumption during adolescence alters the hippocampal response to traumatic brain injury. Biochem. Biophys. Res. Commun..

[34] Lira M., Zamorano P., Cerpa W. (2021). Exo70 intracellular redistribution after repeated mild traumatic brain injury. Biol. Res..

[35] Carvajal F.J., Zolezzi J.M., Tapia-Rojas C., Godoy J.A., Inestrosa N.C. (2013). Tetrahydrohyperforin decreases cholinergic markers associated with amyloid-beta plaques, 4-hydroxynonenal formation, and caspase-3 activation in AbetaPP/PS1 mice. J. Alzheimer’s Dis..

[36] Serrano F.G., Tapia-Rojas C., Carvajal F.J., Hancke J., Cerpa W., Inestrosa N.C. (2014). Andrographolide reduces cognitive impairment in young and mature AbetaPPswe/PS-1 mice. Mol. Neurodegener..

[37] Inestrosa N.C., Carvajal F.J., Zolezzi J.M., Tapia-Rojas C., Serrano F., Karmelic D., Toledo E.M., Toro A., Toro J., Santos M.J. (2013). Peroxisome proliferators reduce spatial memory impairment, synaptic failure, and neurodegeneration in brains of a double transgenic mice model of Alzheimer’s disease. J. Alzheimer’s Dis..

[38] Broadbent N.J., Gaskin S., Squire L.R., Clark R.E. (2010). Object recognition memory and the rodent hippocampus. Learn. Mem..

[39] Ennaceur A. (2010). One-trial object recognition in rats and mice: Methodological and theoretical issues. Behav. Brain Res..

[40] Waldo Cerpa E.L.-E.A.B. (2015). RoR2 functions as a noncanonical Wnt receptor thatregulates NMDAR-mediated synaptic transmission. Proc. Natl. Acad. Sci. USA.

[41] Anderson W.W., Collingridge G.L. (2007). Capabilities of the WinLTP data acquisition program extending beyond basic LTP experimental functions. J. Neurosci. Methods.

[42] Chen M., Lu T.J., Chen X.J., Zhou Y., Chen Q., Feng X.Y., Xu L., Duan W.H., Xiong Z.Q. (2008). Differential roles of NMDA receptor subtypes in ischemic neuronal cell death and ischemic tolerance. Stroke.

[43] Chen G., Chen K.S., Knox J., Inglis J., Bernard A., Martin S.J., Justice A., McConlogue L., Games D., Freedman S.B. (2000). A learning deficit related to age and beta-amyloid plaques in a mouse model of Alzheimer’s disease. Nature.

[44] Toledo E.M., Inestrosa N.C. (2010). Activation of Wnt signaling by lithium and rosiglitazone reduced spatial memory impairment and neurodegeneration in brains of an APPswe/PSEN1DeltaE9 mouse model of Alzheimer’s disease. Mol. Psychiatry.

[45] Dosemeci A., Tao-Cheng J.H., Vinade L., Jaffe H. (2006). Preparation of postsynaptic density fraction from hippocampal slices and proteomic analysis. Biochem. Biophys. Res. Commun..

[46] Hardingham G.E., Bading H. (2002). Coupling of extrasynaptic NMDA receptors to a CREB shut-off pathway is developmentally regulated. Biochim. Biophys. Acta.

[47] Pelkey K.A., Askalan R., Paul S., Kalia L.V., Nguyen T.H., Pitcher G.M., Salter M.W., Lombroso P.J. (2002). Tyrosine phosphatase STEP is a tonic brake on induction of long-term potentiation. Neuron.

[48] Li R., Xie D.D., Dong J.H., Li H., Li K.S., Su J., Chen L.Z., Xu Y.F., Wang H.M., Gong Z. (2014). Molecular mechanism of ERK dephosphorylation by striatal-enriched protein tyrosine phosphatase. J. Neurochem..

[49] Goebel-Goody S.M., Baum M., Paspalas C.D., Fernandez S.M., Carty N.C., Kurup P., Lombroso P.J. (2012). Therapeutic implications for striatal-enriched protein tyrosine phosphatase (STEP) in neuropsychiatric disorders. Pharmacol. Rev..

[50] Hermel E., Gafni J., Propp S.S., Leavitt B.R., Wellington C.L., Young J.E., Hackam A.S., Logvinova A.V., Peel A.L., Chen S.F. (2004). Specific caspase interactions and amplification are involved in selective neuronal vulnerability in Huntington’s disease. Cell Death Differ..

[51] Castonguay D., Dufort-Gervais J., Menard C., Chatterjee M., Quirion R., Bontempi B., Schneider J.S., Arnsten A.F.T., Nairn A.C., Norris C.M. (2018). The Tyrosine Phosphatase STEP Is Involved in Age-Related Memory Decline. Curr. Biol..

[52] Munoz J.J., Tarrega C., Blanco-Aparicio C., Pulido R. (2003). Differential interaction of the tyrosine phosphatases PTP-SL, STEP and HePTP with the mitogen-activated protein kinases ERK1/2 and p38alpha is determined by a kinase specificity sequence and influenced by reducing agents. Biochem. J..

[53] Paul S., Nairn A.C., Wang P., Lombroso P.J. (2003). NMDA-mediated activation of the tyrosine phosphatase STEP regulates the duration of ERK signaling. Nat. Neurosci..

[54] Aizenman E., Lipton S.A., Loring R.H. (1989). Selective modulation of NMDA responses by reduction and oxidation. Neuron.

[55] Patel R., Sesti F. (2016). Oxidation of ion channels in the aging nervous system. Brain Res..

[56] Paula-Lima A.C., Adasme T., Hidalgo C. (2014). Contribution of Ca^2+^ release channels to hippocampal synaptic plasticity and spatial memory: Potential redox modulation. Antioxid. Redox Signal..

[57] Aizenman E. (1995). Modulation of N-methyl-D-aspartate receptors by hydroxyl radicals in rat cortical neurons in vitro. Neurosci. Lett..

[58] Aizenman E., Hartnett K.A., Reynolds I.J. (1990). Oxygen free radicals regulate NMDA receptor function via a redox modulatory site. Neuron.

[59] Carroll R.C., Zukin R.S. (2002). NMDA-receptor trafficking and targeting: Implications for synaptic transmission and plasticity. Trends Neurosci..

[60] Cahill-Smith S., Li J.M. (2014). Oxidative stress, redox signalling and endothelial dysfunction in ageing-related neurodegenerative diseases: A role of NADPH oxidase 2. Br. J. Clin. Pharmacol..

[61] Ghosh N., Ghosh R., Mandal S.C. (2011). Antioxidant protection: A promising therapeutic intervention in neurodegenerative disease. Free Radic. Res..

[62] Lewerenz J., Maher P. (2011). Control of redox state and redox signaling by neural antioxidant systems. Antioxid. Redox Signal..

[63] Von Bernhardi R., Eugenin J. (2012). Alzheimer’s disease: Redox dysregulation as a common denominator for diverse pathogenic mechanisms. Antioxid. Redox Signal..

[64] Huang Y.H., Lin Y., Brown T.E., Han M.H., Saal D.B., Neve R.L., Zukin R.S., Sorg B.A., Nestler E.J., Malenka R.C. (2008). CREB modulates the functional output of nucleus accumbens neurons: A critical role of N-methyl-D-aspartate glutamate receptor (NMDAR) receptors. J. Biol. Chem..

[65] Meyer D.A., Torres-Altoro M.I., Tan Z., Tozzi A., Di Filippo M., DiNapoli V., Plattner F., Kansy J.W., Benkovic S.A., Huber J.D. (2014). Ischemic stroke injury is mediated by aberrant Cdk5. J. Neurosci. Off. J. Soc. Neurosci..

[66] Plattner F., Hernandez A., Kistler T.M., Pozo K., Zhong P., Yuen E.Y., Tan C., Hawasli A.H., Cooke S.F., Nishi A. (2014). Memory enhancement by targeting Cdk5 regulation of NR2B. Neuron.

[67] Tassin T.C., Benavides D.R., Plattner F., Nishi A., Bibb J.A. (2015). Regulation of ERK Kinase by MEK1 Kinase Inhibition in the Brain. J. Biol. Chem..

[68] Yousuf M.A., Tan C., Torres-Altoro M.I., Lu F.M., Plautz E., Zhang S., Takahashi M., Hernandez A., Kernie S.G., Plattner F. (2016). Involvement of aberrant Cdk5/p25 activity in experimental traumatic brain injury. J. Neurochem..

[69] Fan J., Gladding C.M., Wang L., Zhang L.Y., Kaufman A.M., Milnerwood A.J., Raymond L.A. (2012). P38 MAPK is involved in enhanced NMDA receptor-dependent excitotoxicity in YAC transgenic mouse model of Huntington disease. Neurobiol. Dis..

[70] Barria A., Malinow R. (2002). Subunit-specific NMDA receptor trafficking to synapses. Neuron.

[71] Barria A., Malinow R. (2005). NMDA receptor subunit composition controls synaptic plasticity by regulating binding to CaMKII. Neuron.

[72] Groc L., Heine M., Cousins S.L., Stephenson F.A., Lounis B., Cognet L., Choquet D. (2006). NMDA receptor surface mobility depends on NR2A-2B subunits. Proc. Natl. Acad. Sci. USA.

[73] Goebel S.M., Alvestad R.M., Coultrap S.J., Browning M.D. (2005). Tyrosine phosphorylation of the N-methyl-D-aspartate receptor is enhanced in synaptic membrane fractions of the adult rat hippocampus. Brain Res. Mol. Brain Res..

[74] Schumann J., Michaeli A., Yaka R. (2009). Src-protein tyrosine kinases are required for cocaine-induced increase in the expression and function of the NMDA receptor in the ventral tegmental area. J. Neurochem..

[75] Braithwaite S.P., Adkisson M., Leung J., Nava A., Masterson B., Urfer R., Oksenberg D., Nikolich K. (2006). Regulation of NMDA receptor trafficking and function by striatal-enriched tyrosine phosphatase (STEP). Eur. J. Neurosci..

[76] Saavedra A., Ballesteros J.J., Tyebji S., Martinez-Torres S., Blazquez G., Lopez-Hidalgo R., Azkona G., Alberch J., Martin E.D., Perez-Navarro E. (2018). Proteolytic Degradation of Hippocampal STEP61 in LTP and Learning. Mol. Neurobiol..

[77] Cases S., Saavedra A., Tyebji S., Giralt A., Alberch J., Perez-Navarro E. (2018). Age-related changes in STriatal-Enriched protein tyrosine Phosphatase levels: Regulation by BDNF. Mol. Cell. Neurosci..

[78] Goebel-Goody S.M., Wilson-Wallis E.D., Royston S., Tagliatela S.M., Naegele J.R., Lombroso P.J. (2012). Genetic manipulation of STEP reverses behavioral abnormalities in a fragile X syndrome mouse model. Genes Brain Behav..

[79] Kabadi S.V., Faden A.I. (2014). Neuroprotective strategies for traumatic brain injury: Improving clinical translation. Int. J. Mol. Sci..

[80] Chen X.H., Johnson V.E., Uryu K., Trojanowski J.Q., Smith D.H. (2009). A lack of amyloid beta plaques despite persistent accumulation of amyloid beta in axons of long-term survivors of traumatic brain injury. Brain Pathol..

[81] Nakagawa Y., Nakamura M., McIntosh T.K., Rodriguez A., Berlin J.A., Smith D.H., Saatman K.E., Raghupathi R., Clemens J., Saido T.C. (1999). Traumatic brain injury in young, amyloid-beta peptide overexpressing transgenic mice induces marked ipsilateral hippocampal atrophy and diminished Abeta deposition during aging. J. Comp. Neurol..

[82] Smith D.H., Uryu K., Saatman K.E., Trojanowski J.Q., McIntosh T.K. (2003). Protein accumulation in traumatic brain injury. Neuromol. Med..

[83] Yang S.T., Hsiao I.T., Hsieh C.J., Chiang Y.H., Yen T.C., Chiu W.T., Lin K.J., Hu C.J. (2015). Accumulation of amyloid in cognitive impairment after mild traumatic brain injury. J. Neurol. Sci..

[84] Wolf M.S., Bayir H., Kochanek P.M., Clark R.S.B. (2018). The role of autophagy in acute brain injury: A state of flux?. Neurobiol. Dis..

[85] DeWalt G.J., Mahajan B., Foster A.R., Thompson L.D.E., Marttini A.A., Schmidt E.V., Mansuri S., D’Souza D., Patel S.B., Tenenbaum M. (2018). Region-specific alterations in astrocyte and microglia morphology following exposure to blasts in the mouse hippocampus. Neurosci. Lett..

[86] Hall E.D., Wang J.A., Miller D.M. (2012). Relationship of nitric oxide synthase induction to peroxynitrite-mediated oxidative damage during the first week after experimental traumatic brain injury. Exp. Neurol..

[87] Witcher K.G., Bray C.E., Dziabis J.E., McKim D.B., Benner B.N., Rowe R.K., Kokiko-Cochran O.N., Popovich P.G., Lifshitz J., Eiferman D.S. (2018). Traumatic brain injury-induced neuronal damage in the somatosensory cortex causes formation of rod-shaped microglia that promote astrogliosis and persistent neuroinflammation. Glia.

[88] Zhang Q.G., Laird M.D., Han D., Nguyen K., Scott E., Dong Y., Dhandapani K.M., Brann D.W. (2012). Critical role of NADPH oxidase in neuronal oxidative damage and microglia activation following traumatic brain injury. PLoS ONE.

[89] Brun V.H., Otnass M.K., Molden S., Steffenach H.A., Witter M.P., Moser M.B., Moser E.I. (2002). Place cells and place recognition maintained by direct entorhinal-hippocampal circuitry. Science.

[90] Tsien J.Z., Huerta P.T., Tonegawa S. (1996). The essential role of hippocampal CA1 NMDA receptor-dependent synaptic plasticity in spatial memory. Cell.

[91] Nakagawa A., Fujimura M., Kato K., Okuyama H., Hashimoto T., Takayama K., Tominaga T. (2008). Shock wave-induced brain injury in rat: Novel traumatic brain injury animal model. Acta Neurochir. Suppl..

[92] Gao X., Deng P., Xu Z.C., Chen J. (2011). Moderate traumatic brain injury causes acute dendritic and synaptic degeneration in the hippocampal dentate gyrus. PLoS ONE.

[93] Hegde A.N. (2010). The ubiquitin-proteasome pathway and synaptic plasticity. Learn. Mem..

[94] Zhang S., Taghibiglou C., Girling K., Dong Z., Lin S.Z., Lee W., Shyu W.C., Wang Y.T. (2013). Critical role of increased PTEN nuclear translocation in excitotoxic and ischemic neuronal injuries. J. Neurosci. Off. J. Soc. Neurosci..

[95] Kim G.H., Kim J.E., Rhie S.J., Yoon S. (2015). The Role of Oxidative Stress in Neurodegenerative Diseases. Exp. Neurobiol..

[96] Jang S.S., Royston S.E., Xu J., Cavaretta J.P., Vest M.O., Lee K.Y., Lee S., Jeong H.G., Lombroso P.J., Chung H.J. (2015). Regulation of STEP61 and tyrosine-phosphorylation of NMDA and AMPA receptors during homeostatic synaptic plasticity. Mol. Brain.

[97] Herrup K., Yang Y. (2007). Cell cycle regulation in the postmitotic neuron: Oxymoron or new biology?. Nat. Rev. Neurosci..

[98] Inata Y., Kikuchi S., Samraj R.S., Hake P.W., O’Connor M., Ledford J.R., O’Connor J., Lahni P., Wolfe V., Piraino G. (2018). Autophagy and mitochondrial biogenesis impairment contribute to age-dependent liver injury in experimental sepsis: Dysregulation of AMP-activated protein kinase pathway. Faseb J. Off. Publ. Fed. Am. Soc. Exp. Biol..

[99] Hegde A.N., Upadhya S.C. (2011). Role of ubiquitin-proteasome-mediated proteolysis in nervous system disease. Biochim. Biophys. Acta.

[100] Cenini G., Voos W. (2016). Role of Mitochondrial Protein Quality Control in Oxidative Stress-induced Neurodegenerative Diseases. Curr. Alzheimer Res..

[101] Bezprozvanny I., Hayden M.R. (2004). Deranged neuronal calcium signaling and Huntington disease. Biochem. Biophys. Res. Commun..

[102] Cardoso S., Seica R.M., Moreira P.I. (2017). Mitochondria as a target for neuroprotection: Implications for Alzheimer s disease. Expert Rev. Neurother..

[103] Kokiko-Cochran O.N., Godbout J.P. (2018). The Inflammatory Continuum of Traumatic Brain Injury and Alzheimer’s Disease. Front. Immunol..

[104] Johnson V.E., Stewart W., Smith D.H. (2012). Widespread tau and amyloid-beta pathology many years after a single traumatic brain injury in humans. Brain Pathol..

[105] Smith D.H., Chen X.H., Nonaka M., Trojanowski J.Q., Lee V.M., Saatman K.E., Leoni M.J., Xu B.N., Wolf J.A., Meaney D.F. (1999). Accumulation of amyloid beta and tau and the formation of neurofilament inclusions following diffuse brain injury in the pig. J. Neuropathol. Exp. Neurol..

