# Supplementary material for: Regulation of Phosphorylated State of NMDA Receptor by STEP61 Phosphatase after Mild-Traumatic Brain Injury: Role of Oxidative Stress
Source: Antioxidants (Basel). 2021 Oct 5;10(10):1575. doi: 10.3390/antiox10101575 (PMC8533270; doi:10.3390/antiox10101575)
Supplement: Supplementary file 1 [file antioxidants-10-01575-s001.zip › antioxidants-1318629-supplementary.pdf]

Supplementary Material

Supplementary Table 1. List of antibodies used for western-blot analysis and immunofluorescence studies.

Antibody	Company	Dilution
GFAP	Sigma-Aldrich	1:500 for IF
Iba-1	Sigma-Aldrich	1:500 for IF
4-Hydroxynonenal (4-HNE)	USBiological	1:200 for IF
8-hydroxy-2'-deoxyguanosine (8-OHdG)	Santa Cruz Biotechnology	1:200 for IF
Actin	Novus Biological	1:1000 for WB
Tubulin	Santa Cruz Biotechnology	1:1000 for WB
GAPDH	Santa Cruz Biotechnology	1:1000 for WB
STEP	Novus Biological	1:1000 for WB
GluN2B	Sigma-Aldrich	1:1000 for WB
GluN2B p-1472	Cell Signaling Technology	1:1000 for WB
GluN2B p-1336	Invitrogen	1:1000 for WB
p-CREB s113	Novus Biological	1:1000 for WB
PSD-95	Santa Cruz Biotechnology	1:1000 for WB
Synaptophysin	Santa Cruz Biotechnology	1:1000 for WB
ERK	ThermoFisher Scientific	1:1000 for WB
p-ERK	ThermoFisher Scientific	1:1000 for WB

[1] The specificity, isotype, clone number, and commercial source of the antibodies used throughout the study are indicated. All antibodies were used according to the manufacturer's instructions, indicating the dilution and technique used for the antibodies. IF, immunofluorescence; WB, western-blot.

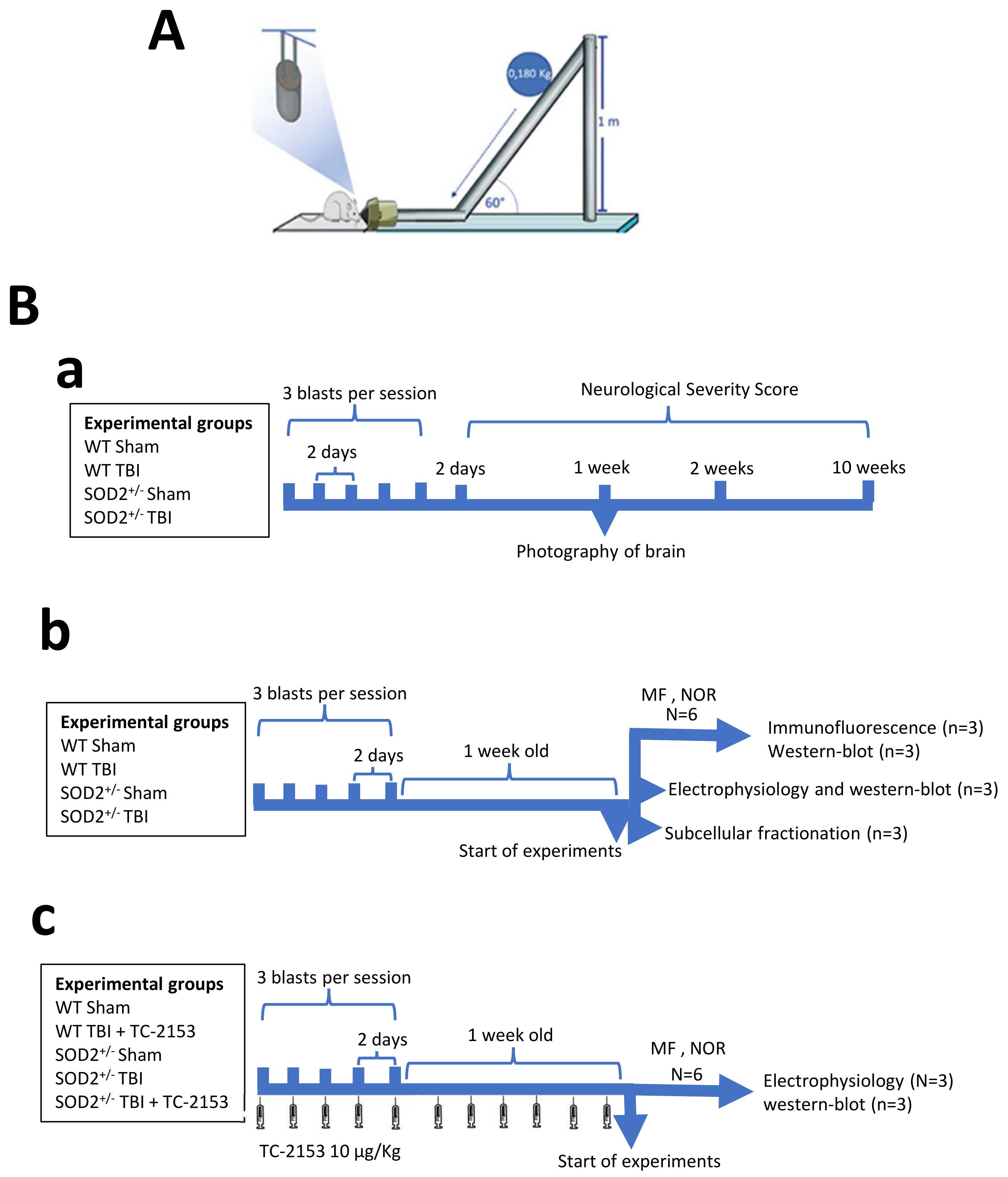
A
0,180 Kg
1 m
60°
B
a
Experimental groups
WT Sham
WT TBI
SOD2+/- Sham
SOD2+/- TBI
3 blasts per session
2 days
2 days
Neurological Severity Score
1 week
2 weeks
10 weeks
Photography of brain
b
Experimental groups
WT Sham
WT TBI
SOD2+/- Sham
SOD2+/- TBI
3 blasts per session
2 days
1 week old
MF , NOR
N=6
Immunofluorescence (n=3)
Western-blot (n=3)
Electrophysiology and western-blot (n=3)
Subcellular fractionation (n=3)
Start of experiments
c
Experimental groups
WT Sham
WT TBI + TC-2153
SOD2+/- Sham
SOD2+/- TBI
SOD2+/- TBI + TC-2153
3 blasts per session
2 days
1 week old
MF , NOR
N=6
Electrophysiology (N=3)
western-blot (n=3)
TC-2153 10 µg/Kg
Start of experiments

Supplementary Figure 1: Experimental design of mild traumatic brain injury model. A. Apparatus for the application of frontal impact closed head traumatic brain injury. This schematic diagram depicting the essential elements of the injury apparatus, illustrating the key feature of a ball rolling down rails and striking the coupling arm attached to the anterior part of the mouse's face. **B**. Experimental procedure of traumatic brain injury induction. Mice were subject to mild and repetitive injury induction for 5 sessions each two days. The session consists of 3 consecutive blasts. The animals were separated into three experimental sets. The first experimental set was formed only from WT and $SOD2^{+/-}$ animals to generate control tests on the TBI induction model. In this group, the NSS was evaluated at 2 days, 1, 3, and 10 weeks after the last trauma induction session (Supplementary Figure 1Ba). A second experimental set was formed by WT and $SOD2^{+/-}$ animals sham and subjected to TBI. This group was used to measure the consequences of TBI at the level of memory, electrophysiology, markers of inflammation and oxidative stress, and the signaling mechanisms involved in neurodegeneration. Testing began 1 week after the last TBI induction session (Supplemental Figure 1Bb). The third experimental set was used to test the effect of the $STEP_{61}$ inhibitor in mice after TBI. Mice were treated with TC-2153 (10 mg/Kg) 2 hours before the TBI induction session. In this experimental group, the animals were subjected to behavioral, electrophysiological, and biochemical tests 1 week after the last trauma induction session, during this rest week, the inhibitor treatment was continued under the same conditions indicated above (Figure Supplementary 1Bc; MF, memory flexibility test; NOR, novel object recognition test).

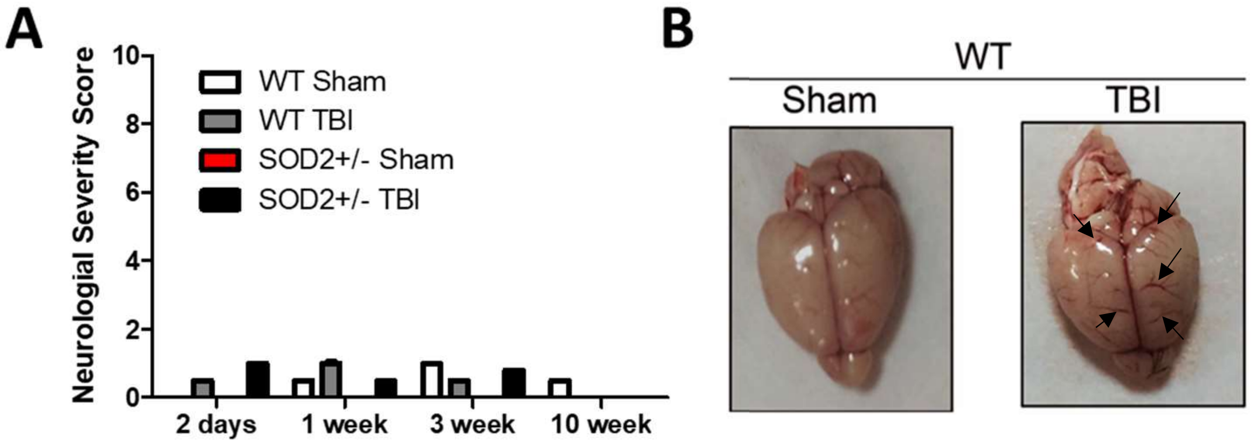

Supplementary Figure 2. Neurologic features after TBI induction. A. Neurobehavioral status was evaluated by the Neurological Severity Score (NSS). NSS was evaluated on 2 days and 1, 3, and 10 weeks following the last session of injury. Task and points of NSS are in supplementary table 2. Over six points in the NSS is considerate neurological deficits**. B.** Photograph of mouse brain 1 week after TBI induction, showing subarachnoid hemorrhage in all brain (Black arrows).

Supplementary table 2: Neurological function scoring system

Task	NSS
Presence of mono- or hemiparesis	1
Inability to walk on a 3 cm wide beam	1
Inability to walk on a 2 cm wide beam	1
Inability to walk on a 1 cm wide beam	1
Inability to balance on a 1 cm wide beam	1
Inability to balance on a round wide beam (0,5 cm wide)	1
Failure to exit a 30 cm diameter circle (for 2 min)	1
Inability to walk straight	1
Loss of startle behavior	1
Loss of seeking behavior	1
**Maximum total**	10
